# Relationships between Premenstrual Syndrome (PMS) and Diet Composition, Dietary Patterns and Eating Behaviors

**DOI:** 10.3390/nu16121911

**Published:** 2024-06-17

**Authors:** Paulina Oboza, Natalia Ogarek, Mariusz Wójtowicz, Tahar Ben Rhaiem, Magdalena Olszanecka-Glinianowicz, Piotr Kocełak

**Affiliations:** 1 Pathophysiology Unit, Department of Pathophysiology, Faculty of Medical Sciences, Medical University of Silesia, 40-055 Katowice, Poland; 2 Clinical Department of Gynecology and Obstetrics, Faculty of Medical Sciences in Zabrze, Medical University of Silesia, 40-055 Katowice, Poland; 3 Clinical Department of Obstetrics, Gynecology and Gynecological Oncology in Kędzierzyn-Koźle, Faculty of Medicine, Medical University, 45-052 Opole, Poland; 4 Health Promotion and Obesity Management Unit, Department of Pathophysiology, Faculty of Medical Sciences in Katowice, Medical University of Silesia, 40-055 Katowice, Poland

**Keywords:** premenstrual syndrome, diet composition, dietary patterns, eating behaviors

## Abstract

Premenstrual Syndrome (PMS) is a disorder between gynecology and psychiatry which includes cognitive, affective, and somatic symptoms from mild to severe. The most severe form of PMS is premenstrual dysphoric disorder (PMDD) and it is considered a form of depressive disorder. An association between diet composition and the occurrence of PMS and its severity have been suggested. As such, this manuscript discusses the relationships between diet composition, dietary patterns and eating behaviors, and PMS. PubMed, Embase, Cochrane, and Web of Science databases were searched for related studies up to 18 January 2024. A text search with the following keywords singly or in combination was conducted: “Premenstrual syndrome”, “Nutrition”, “Diet composition”, “Dietary patterns”, and “Eating behaviors”. Studies published so far showed that low intake of simple carbohydrates, fats, salt, and alcohol, and high of fresh, unprocessed foods rich in B vitamins, vitamin D, zinc, calcium, and omega-3 fatty acids may help prevent the onset of PMS and reduce the severity of its symptoms. However, further studies are needed to formulate definitive recommendations for the use of vitamins, micronutrients and other dietary ingredients supplementation in women with PMS to improve functioning, overall well-being, and physical health. Large, randomized, double-blind clinical trials across diverse populations are necessary to formulate clear recommendations for supplementation in women with PMS.

## 1. Introduction

There is no universally accepted definition of premenstrual syndrome (PMS). It consists of both gynecological and psychiatric domains. The existing definitions remain incomplete and fragmented [1,2]. One commonly accepted definition characterizes PMS as a predictable, cyclic symptom cluster occurring in the luteal phase of the menstrual cycle, significantly disrupting various aspects of daily life [3]. The diagnostic criteria for PMS from the American College of Obstetricians and Gynecologists (ACOG) include the emergence of symptoms within five days before menstruation in at least three consecutive menstrual cycles and their resolution within four days after menstruation commences [2]. The ACOG proposes a definition of PMS to include both physical and psychiatric symptoms. However, this definition does not include the potential exacerbation of pre-existing disorders such as depression or panic disorder. In turn, the American Psychiatric Association (APA) definition of PMS focuses solely on psychiatric symptoms and explicitly excludes a diagnosis if the symptoms are exacerbations of another disease. Similarly, the World Health Organization (WHO) definition focuses on mental symptoms and classifies PMS in the International Classification of Diseases (ICD)-10 as Premenstrual Tension Syndrome. These different criteria create difficulties in diagnosing PMS. Moreover, it may be the cause of an underestimation of the severity of the disease. While rigorous use of the DSM-V criteria may result in a lack of diagnosis and inadequate treatment of women with significant dysfunction related to PMS [4]. Nevertheless, despite the inconsistencies in the PMS diagnostic criteria, treatment guideline recommendations include lifestyle changes, nonpharmacological interventions, and pharmacological, and surgical treatment [5,6,7].

A meta-analysis assessing the epidemiology of PMS showed a cumulative incidence of approximately 47.8% among women worldwide (95% CI: 32.6–62.9) [8]. Moreover, other meta-analyses revealed an overall prevalence of PMS of 48%, including 40% in Europe, 85% in Africa, 46% in Asia, and 60% in South America [9]. Furthermore, it has been shown that the pooled prevalence of PMS in Africa was 46.98% (95% CI: 28.9–65.06%) [10]. In addition, studies conducted in Europe showed the occurrence of PMS in 32.1% of Bulgarian and 31.9% of Italian women, and PMDD in 3.3% of Bulgarian women [11,12]. In contrast, a significantly lower occurrence of PMS 7.0% was observed among Korean women, with a gradual increase of approximately 1% per year during 8-year follow-up [13]. A higher occurrence of PMS was shown among young Arab women compared to European women. Seventy-seven per cent (77.7%) of Egyptian women, (72.9%) of Jordanian women, and (66.3%) of Syrian women, with PMDD observed in 40%, 34.7%, and 28.2% respectively [8]. Furthermore, in a cohort of Turkish women aged 18–25 years, PMS was found in 49.2%, and PMDD in 48.0% [14].

PMS symptoms include increased appetite, weight gain, abdominal and back pain, headaches, breast tenderness, nausea, constipation, anxiety, irritability, fatigue, mood swings, and tearfulness and the severity of symptoms may vary [15]. Premenstrual Dysphoric Disorder (PMDD) is one of the most severe forms of PMS. PMDD is included in the DSM-5, in the spectrum of depressive disorders. It is characterized by cognitive, affective, and somatic symptoms [2,16]. 

The pathogenesis of PMS remains unclear. Changes in sex steroid levels, especially progesterone, and in central neurotransmitters including serotonin, gamma-aminobutyric acid (GABA), glutamate, and beta-endorphins, play an important role in PMS pathogenesis [17]. In addition to family predispositions, diet and nutritional deficiencies can participate in the development of PMS [18]. It has also been shown that an increase in BMI of 1 kg/m^2^ is associated with a 3% increase in the risk of PMS development [19]. However, an association between PMS and being underweight was also shown (HR = 1.21, 95% CI 1.10–1.25) [20]. 

The associations between diet composition and the occurrence of PMS and its severity were suggested in some studies. In addition, changes in dietary patterns and eating behaviors were observed in women with PMS. Therefore, the aim of this manuscript is to analyze the available data on the relationship between PMS and diet composition, dietary patterns, and eating behaviors.

## 2. Changes in Energy and Macronutrient Intake during the Menstrual Cycle

Total energy intake fluctuated during the menstrual cycle. The highest intake was observed during the luteal phase in animal models [21] and human studies [22,23]. These changes can be explained by the different effects of estradiol and progesterone on hunger and appetite. Estradiol directly inhibits food intake and increases energy expenditure [24], while progesterone stimulates food intake [25]. Estradiol acts directly on anorexigenic and orexigenic neurons in the hypothalamus and brainstem [19,26] and indirectly increases the release of gastrointestinal hormones, including cholecystokinin (CCK), glucagon-like peptide-1 (GLP-1), and other hormones such as insulin and leptin, which stimulate the release of neurotransmitters responsible for the feeling of satiety and inhibit the release of neurotransmitters responsible for the feeling of hunger in the arcuate nucleus of the hypothalamus [26,27,28,29]. Furthermore, estradiol inhibits the release of the hunger-stimulating hormone ghrelin in the stomach [30]. 

Studies assessing changes in energy intake during the menstrual cycle are inconclusive. Some show an increase in intake from 87 to 500 kcal during the luteal phase [31,32], while others found no significant changes [33,34,35]. Interestingly, it has been observed that estradiol levels influence women’s perceptions of food [24,36]. Lower estradiol and higher progesterone levels during the luteal phase were associated with increased reactivity to high-energy food pictures in studies performed using EEG and functional magnetic resonance [36,37,38,39]. In addition, more frequent food cravings, binge eating, and increased fat or carbohydrate intake were observed during the luteal phase [40,41,42,43,44].

## 3. PMS and Dietary Composition and Patterns

The relationship between PMS, dietary composition, and dietary patterns seems to be bidirectional. However, the results of studies assessing these relationships are inconclusive. A recently published study showed no association between dietary components, except for copper intake and PMS [45]. Moreover, no association between PMS and the intake of cereals, dairy products, caffeine, fat, sweets, vegetables, fruits, spices, or pickles was shown [46]. Furthermore, Houghton et al. [47] observed no correlation between fiber, carbohydrate, and protein intake and PMS [47]. While, another study found a significant premenstrual increase in fat and simple carbohydrates, as well as a decrease in protein intake in women with PMS [48]. Furthermore, a significant inverse association between PMS severity and fish and seafood consumption was observed [46]. Moreover, positive correlations were observed between consuming a high-energy diet rich in fat, sugar, and salt and with severity of physical symptoms of PMS. Fruit consumption was associated with a decreased risk of psychological symptoms of PMS [49]. In addition, a relationship between a diet rich in red and processed meat, fast food, vegetable oil, mayonnaise, deep-fried foods, salty snacks, refined grains, sugar and soft drinks, high-fat dairy products, spices, and fried potatoes and an increased risk of developing PMS symptoms, was observed [50]. 

It has also been suggested that women with PMS are more sensitive to hormonal fluctuations during the menstrual cycle, resulting in higher consumption of simple carbohydrates during the premenstrual period [36]. Simple carbohydrate intake may be a counterregulatory mechanism for decreased mood because it increases serotonin and dopamine release in the brain [51]. Mood swings are a common symptom of PMS. The associations between serotonin release in the brain and mood regulation were found [52]. Carbohydrates increase the availability of tryptophan, a precursor of serotonin. A sharply lowering blood glucose level may be the cause of irritability. Thus, changes in blood glucose levels can shape the relationship between carbohydrate consumption and mood fluctuations [53]. However, a recently published study has shown a similar simple carbohydrate and fiber intake in women with and without PMS [45].

It should be noted that the current studies assessing the main dietary factors provide inadequate data. Most studies have focused on the assessment of the potential effects of selected dietary components, which limits the accuracy of the conclusions. Importantly, PMS *per se* may affect food choices, and these choices can influence the worsening or relief of symptoms. Therefore, the data so far permits the conclusion that there is a relationship between the composition of diet and PMS but does not allow us to establish a certain cause-and-effect relationship.

### 3.1. Macronutrient Intake and PMS

Studies assessing the association between macronutrient intake and PMS have been inconclusive. Some studies have shown a positive association between PMS symptoms and a diet rich in simple carbohydrates, fried foods, and alcohol and a negative association between PMS symptoms and a diet rich in vegetables, fruits, and fiber [19,47,50,54,55]. Another study found no association between fiber and carbohydrate intake, except for maltose, and PMS development. The highest intake of maltose was associated with a 45% increased risk of developing PMS after adjusting for BMI, smoking, and other factors [56].

A large cohort study found no association between fat intake and PMS risk [57]. Another study showed a negative association between high stearic acid intake and PMS development [56]. Furthermore, no correlation between protein intake and PMS was found [58]. The association between micronutrient intake and PMS is shown in Table 1.

In summary, assessing the relationship between the occurrence of PMS, the severity of its symptoms, and the consumption of macronutrients is very difficult, which may result in large discrepancies in the results of published studies. These relationships may also be temporal. In diet composition analyses, it is impossible to reliably assess the long-term intake of individual macronutrients. Moreover, it should be emphasized that all the tools used to analyze diet composition have numerous limitations, including respondents’ memory and truthfulness. Therefore, based on the available data, it is difficult to formulate recommendations regarding the consumption of macronutrients to alleviate the severity of PMS symptoms.

### 3.2. Micronutrient Intake and PMS 

Several hypotheses have been proposed regarding the effect of micronutrient deficiencies on the development of PMS.

One study suggested that PMS is a clinical manifestation of calcium deficiency. This hypothesis may be supported by changes in calcium concentration during the menstrual cycle and the relationship between calcium homeostasis and affective disorders [59]. Moreover, significantly lower calcium and magnesium levels have been observed in women with PMS. Furthermore, calcium, magnesium, and potassium intakes were lower in women with PMS than without PMS [60]. In addition, supplementation with 1200 mg of calcium carbonate daily during three menstrual cycles in women with PMDD reduced the severity of psychological and physical symptoms by 48% [61]. 

Another hypothesis links PMS with iron deficiency. It has been shown that a higher intake of iron, especially non-heme iron, significantly reduces the risk of development of PMS [62]. Moreover, PMS symptoms, including confusion, headaches, and nausea, are less common in women with a genetically increased risk of iron overload [63]. 

In addition, a randomized clinical trial (RCT) showed that supplementation with 220 mg of elemental zinc daily for 24 weeks reduced the severity of PMS symptoms and improved quality of life compared with the placebo group [64,65]. Most of these studies were conducted among Arab women. The associations between micronutrient intake and PMS are shown in Table 2.

### 3.3. Vitamin Intake and PMS

An inverse association was observed between thiamine and riboflavin intake and PMS. However, supplementation did not significantly reduce the severity of PMS symptoms [66]. 

Studies assessing the effect of vitamin B6 supplementation on PMS symptoms are inconclusive. One study showed no difference in PMS symptom severity between the group supplemented with vitamin B6 (80 mg/day) and the group supplemented with broad-spectrum micronutrient formulas during three menstrual cycles. Complete remission of PMS symptoms was observed in 72% of the group supplemented with micronutrients and 60% of the group supplemented with vitamin B6 [67]. A meta-analysis of 12 case-control studies, including 586 women with PMS supplemented with vitamin B6 and 602 receiving a placebo, found a significant improvement in both the physical and psychological symptoms of PMS in women supplemented with vitamin B6 [68]. 

Furthermore, some trials have shown that supplementation with 80 mg of thiamine daily for two menstrual cycles decreased the severity of PMS symptoms compared with placebo [69]. Moreover, another study found that daily supplementation with 100 mg thiamine and 500 mg calcium carbonate reduced the severity of PMS symptoms more than supplementation with 100 mg thiamine, 500 mg calcium carbonate, or placebo [70].

There are also associations between vitamin D insufficiency and the risk of developing PMS [71] and the severity of its symptoms [72]. However, vitamin D intake did not affect the risk of developing PMS [73,74]. Supplementation with 50,000 IU/week of vitamin D decreased the incidence of several symptoms of PMS, including back pain and a tendency to cry easily, as well as the severity of dysmenorrhea in adolescents [75]. Moreover, vitamin D supplementation (200,000 IU initially, followed by 25,000 IU every 2 weeks) for 4 months decreased mood symptoms related to PMS in young women with severe vitamin D insufficiency [76]. The effect of vitamin D supplementation on the reduction of the severity of PMS symptoms has also been confirmed in other studies [77,78,79], systematic reviews, and meta-analyses of 16 studies (5 interventional and 11 observational) which included 4946 women [80]. The associations between vitamin intake and PMS are shown in Table 3.

### 3.4. Other Nutrients and PMS

A randomized, placebo-controlled, double-blind clinical trial involving 40 women with PMS showed that supplementation with lecithin-phosphatidylserine (400 mg daily) and phosphatidic acid complex (400 mg daily) for three menstrual cycles significantly reduced the severity of both physical and psychological symptoms [81]. 

A meta-analysis of eight RCTs found that omega-3 fatty acids may reduce the severity of PMS, but its efficacy depends on the duration of use [82]. While few studies have explored the relationship between inflammation, oxidative stress, and PMS, existing data remains limited [83]. Furthermore, decreased estradiol levels may contribute to the development of inflammation exacerbating premenstrual symptoms such as menstrual pain, mood changes, and increased bleeding [84]. Omega-3 fatty acids have anti-inflammatory properties related to competitive interactions with arachidonic acid as a substrate for cyclooxygenases and 5-lipoxygenases. Especially eicosatetraenoic acid and docosahexaenoic acid reduce inflammation by inhibiting leukocyte chemotaxis, regulating the expression of adhesion molecules, modulating leukocyte-endothelial adhesive interactions, suppressing eicosanoid production, and inhibiting the synthesis of pro-inflammatory cytokines [85,86,87]. Thus, omega-3 fatty acids intake may alleviate PMS symptoms related to inflammation.

The association between caffeine and caffeinated drink consumption and PMS has also been assessed, but the results are inconclusive. Some studies showed a strong positive association between caffeine and caffeinated drink consumption and PMS severity [82,87,88,89,90,91]. However, other studies have not found these associations [92,93]. Moreover, the prospective Nurses’ Health Study II found that highly caffeinated coffee consumption was not associated with the risk of the development of PMS or its specific symptoms, such as breast tenderness [94]. 

It has also been observed that tryptophan supplements and complex carbohydrate-enriched drinks significantly decreased the severity of PMS symptoms compared to placebo [57,95]. 

The associations between intake of other nutrients and PMS are shown in Table 4.

## 4. PMS and Eating Behaviors

The relationship between eating habits and PMS is complex. In a study of 383 adolescents, PMS symptoms were found in 55.9% of participants. Disordered eating was significantly more common in the PMS group than in the non-PMS group. Moreover, emotional and uncontrolled eating scores were higher in the PMS group [96]. Another study showed a significantly higher EAT-26 score and overall prevalence of eating disorders in the group with PMDD than in the group with moderate-to-severe, mild, or no PMS symptoms [97]. It has also been suggested that physical and psychological PMS symptoms are associated with a higher risk of developing eating disorder symptoms [98,99]. In addition, among women with binge eating symptoms, the occurrence of moderate and severe physical and psychological PMS symptoms was significantly more common, while no association between binge eating disorders and either PMS or PMDD was found. However, a more than 7-fold increased odds ratio of developing bulimia nervosa was associated with PMDD, and a more than 2-fold increased odds ratio with PMS [100]. In most studies, self-reporting surveys or diaries were used, and data were analyzed retrospectively, which may introduce potential bias and discrepancies. Furthermore, some studies failed to assess co-existing psychiatric disorders and used supplements or vitamins that may influence PMS symptoms. Moreover, the menstrual cycle phases were not considered. The symptoms of eating disorders may differ between menstrual cycle phases [97]. Additionally, any type of birth control used that may influence eating behavior and PMS symptoms and the timeframe of their use were not analyzed. Considering these limitations, it is impossible to draw definitive conclusions regarding cause and effect relationship. Thus, there is a critical need for large-scale, longitudinal studies in both clinical settings and the general population to clarify the relationship between PMS and eating disorders. 

## 5. Managing PMS

**Figure 1:**
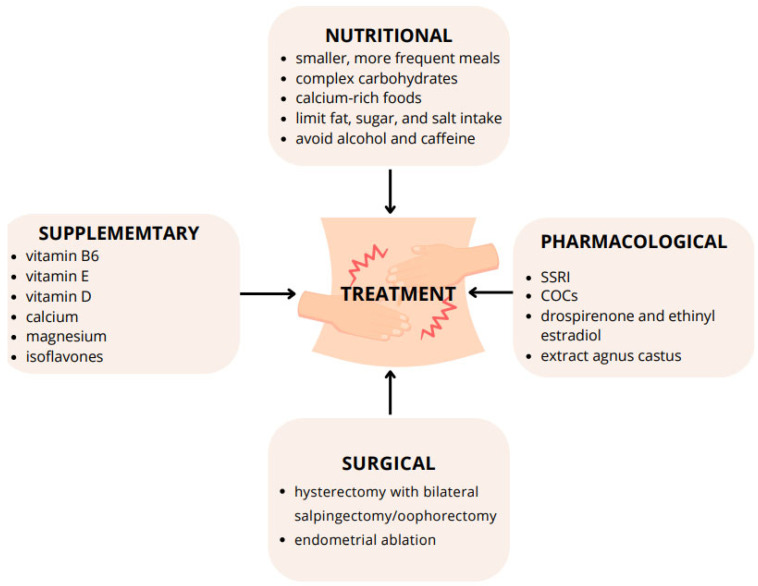
Potential treatment options for women with PMS. Abbreviations: SSRI—selective serotonin reuptake inhibitors, COCs—combined oral contraceptives.

The primary goal of PMS treatment is to alleviate symptoms and minimize their impact on daily activities. PMS treatment should be individualized according to the patient’s symptom profile and should include nonpharmacological and pharmacological interventions. Non-pharmacological interventions include cognitive behavioral therapy [101] and lifestyle changes comprising nutritional therapy [102]. The current guidelines [2,4] for managing PMS do not include individualization of therapy depending on the severity of symptoms and hormonal profile except for surgical treatment. Potential treatment options for women with PMS are shown in Figure 1.

### 5.1. Nonpharmacological Treatment 

#### 5.1.1. Nutritional Treatment

Nutritional treatment is an important component of the non-pharmacological treatment of PMS. This involves the implementation of dietary modifications. It has also been suggested that the involvement of a multidisciplinary healthcare team, including a dietitian, can be beneficial [5]. Researchers at the Mayo Clinic emphasize the importance of not only the composition of the diet but also eating smaller and more frequent meals to alleviate PMS symptoms such as bloating and feelings of fullness [6]. Similarly, the ACOG stressed the importance of meal frequency, suggesting the consumption of six small meals instead of three larger meals. This dietary pattern may help to maintain stable blood glucose levels and potentially reduce PMS symptoms [103]. Consistent guidelines recommend a diet rich in complex carbohydrates, including fruits, vegetables, and whole grains. Such dietary choices can prevent mood fluctuations and food cravings commonly associated with PMS. Moreover, the diet should contain calcium-rich foods, such as yoghurt and leafy greens [6]. In turn, fat, sugar, and salt intake should be limited because excessive consumption promotes bloating and fluid retention [103,104]. In addition, the National Association for Premenstrual Syndrome (NAPS) recommends limiting alcohol and caffeine consumption [6,103,104].

#### 5.1.2. Use of supplements

The Royal College of Obstetricians and Gynecologists, as first-line treatment, for PMDD recommends vitamin B6 supplementation, despite the low level of evidence for its effectiveness [102]. However, high B6 doses may promote the development of peripheral neuropathy [105]. The NAPS recommend vitamin B6 for treating mild-to-moderate PMS, with a maximum daily intake of 50 mg under the supervision of a primary care physician. However, available evidence supporting its efficacy remains insufficient. Furthermore, NAPS suggest the daily use of 1 g calcium and 10 µg vitamin D3, especially for migraine treatment. ACOG recommends supplementation of 1.2 mg calcium daily to alleviate both physical and psychological PMS symptoms, especially reducing water retention and breast tenderness [105,106]. The Mayo Clinic highlights the supplementation of calcium, magnesium, vitamin E, vitamin B6, and herbal remedies but underscores the lack of conclusive evidence supporting their effectiveness [6]. Further studies are necessary to assess the effectiveness of dietary recommendations and refine treatment protocols.

Preliminary results suggest that regular magnesium supplementation at 250 mg daily decreases the severity of PMS symptoms. In addition, some data have indicated the benefits of using isoflavones [106].

The efficacy of the fruit extract of *agnus castus* in reducing the severity of irritability and mood swings related to PMS has also been found [106,107]. However, no comparative studies with SSRIs and oral contraceptive pills have been conducted.

Due to the inadequacy of sufficient data and the unpredictability of the treatment’s efficacy or the substantial shortcomings in the study’s methodology, the use of supplements in the treatment of PMS should be considered as a complementary and not a primary option [3,7].

#### 5.1.3. Cognitive Behavior Therapy

Cognitive behavioral therapy (CBT) is a therapeutic method that focuses on correcting maladaptive thoughts, behaviors, and emotions that cause distress and impair daily functioning [104]. One meta-analysis [108] of five randomized controlled trials (RCTs) of CBT in women with PMS found significant reductions in symptoms of anxiety and depression, although the quality of the trials was rated as low due to weaknesses in study design and implementation and potential reporting bias. However, the majority of evidence supports the effectiveness of CBT for both PMS and PMDD [109]. Moreover, a meta-analysis revealed that CBT is equally effective as antidepressant medications in treating PMS and PMDD, suggesting that combining therapies may result in better outcomes [110].

#### 5.1.4. Lifestyle Modification

Lifestyle modifications include regular physical activity, avoidance of stressful situations, and maintenance of healthy sleep patterns, particularly during the premenstrual period. Knowledge about the beneficial effects of physical activity on health justifies its recommendation in the treatment of PMS [103]. However, the quality of studies showing the positive impact of physical activities including swimming [111], pilates [112] or aerobic exercise [113] on PMS symptoms is limited. Although a meta-analysis of 7 RCT has also shown benefits [114], the variability of these studies limits their reliability. In turn, in a group of 106 young women with PMS divided into three subgroups (diet, aerobic exercise, and control) it was found that both 3 months of diet or aerobic exercise reduced PMS symptoms and dysmenorrhea intensity [115].

### 5.2. Pharmacological Treatment 

#### 5.2.1. Selective Serotonin Reuptake Inhibitors 

Although the mechanism of action remains unclear [116], the gold standard pharmacotherapy for PMDD is treatment with selective serotonin reuptake inhibitors (SSRIs), administered continuously or only during the luteal phase of the menstrual cycle [117]. It is considered that SSRIs modulate the synthesis of allopregnanolone, although their mechanism of action in this regard is unknown [117,118]. One open-label trial assessed treatment with sertraline in PMDD and demonstrated alterations in total peripheral allopregnanolone levels [118]. The beneficial effects of SSRIs have been confirmed in a meta-analysis of 19 RCTs involving 2964 women with PMS and/or PMDD, and no SSRI was superior to the others [119]. However, the side effects related to SSRI use, including sexual dysfunction, suicidal ideation, and insomnia, may reduce their beneficial effects [120,121,122].

#### 5.2.2. Combined Oral Contraceptives 

Combined oral contraceptives are an effective therapy for physical symptoms associated with the menstrual cycle, such as menorrhagia, dysmenorrhea, and gastrointestinal disturbances. However, the data assessing their effect on PMS affective symptoms are inconclusive [123]. Different combinations of hormones, doses, and use times further confound the data. The US Food and Drug Administration (FDA) approved a combination of drospirenone and ethinyl estradiol for the treatment of PMDD [124]. Combined contraceptive pills containing different gestagens and ethinyl estradiol are recommended for treating PMS and PMDD [122]. However, combined oral contraceptive pills are not effective in reducing depressive symptoms [125]. Moreover, they are contraindicated in women with an increased risk of venous thrombosis and breast cancer [126]. The potential side effects include those commonly reported (e.g., headache, metrorrhagia, menorrhagia, acne, intermenstrual vaginal bleeding, decreased libido, mood swings) [127] and those less frequent but serious (thrombotic events [128], an increase in the risk of breast cancer [129]), may be a cause of noncompliance and the decrease of effectiveness.

### 5.3. Surgical Treatment

Hysterectomy with bilateral salpingectomy/oophorectomy is recommended for patients aged 40 years and over, after confirming the lack of effects of conservative treatment [130]. This surgery results in premature menopause and its consequences [131]. Therefore, hormone replacement therapy is necessary to prevent increased cardiovascular risk, osteoporosis [132], depressive and anxiety symptoms and adverse effects on sexual health [133], and cognitive decline [134]. Another option is an invasive endometrial ablation procedure [135]. Nevertheless, the data assessing the effect of surgical treatment on PMS are scarce and further research is required to substantiate these preliminary findings.

### 5.4. Future Therapies

As was mentioned above, one hypothesis concerning the etiology of PMS is the disrupted regulation of GABA receptors. Therefore, one strategy for treating PMS and PMDD may be to regulate the action of allopregnanolone (ALLO) on GABA receptors. Initial studies assessing selective progesterone receptor modulators (SPRMs), especially mifepristone, in the treatment of the symptoms of severe PMS, did not confirm their effectiveness. However, a recent study confirmed the effectiveness of ulipristal acetate (second-generation SPRMs) in the treatment of the emotional and behavioral symptoms of PMDD. Moreover, current studies’ endeavors in the advancement of treatment methods for PMDD are primarily oriented towards achieving stabilization of ALLO signaling. Dutasteride, an inhibitor of 5-alpha-reductase, responsible for the conversion of progesterone to ALLO, is one such candidate. In addition, sepranolone, an allosteric modulator of the GABA-A receptor, is under investigation [136].

### 5.5. Interactions

The components of the treatment of PMS may interact in a complex manner. It has been suggested that the use of COCs may decrease the concentrations of various nutritional ingredients including riboflavin, pyridoxine, folacin, vitamin B12, ascorbic acid, and zinc, and potentially increase the levels of vitamin K, iron, and copper [137,138]. Other studies show a correlation between OC use and reduced levels of vitamin B12 and vitamin B6 [139]. A change in vitamin D levels associated with oral estrogens has also been found [140]. Thus, some data suggest that dietary management may not be effective in reducing PMS symptoms [141]. It has also been shown that high vitamin B6 intake may impair the efficacy of antidepressants [142]. While, magnesium and calcium supplements may affect the bioavailability of antibiotics, including fluoroquinolone [143]. Herbal preparations may increase menstrual bleeding, gastrointestinal symptoms [144] and excessive sleepiness [145]. Thus, simultaneous use of supplements and pharmacological therapies may increase the risk of adverse effects. Moreover, SSRIs such as paroxetine, fluoxetine, and sertraline used in PMS treatment can alter bleeding parameters, potentially exacerbating side effects, for instance, following surgical interventions [146].

### 5.6. Individualized Nutritional Therapy

Considering the reciprocal relationship between diet and PMS, a personalized plan is essential, involving an analysis of patient dietary patterns and macronutrients and micronutrient intake to identify shortcomings and recommend beneficial changes.

It has been suggested that the intake of foods rich in vitamin B and magnesium intensifies the duration of migraine headaches related to PMS [147]. Furthermore, increasing calcium and magnesium intake has the potential to alleviate the effect of mood swings and bloating [6]. Similarly, omega-3 fatty acids, vitamin B6, and vitamin D supplementation can reduce inflammation, stabilize mood, and regulate hormones [85]. While reducing simple carbohydrates may prevent blood glucose level fluctuations and related to exacerbation of irritability or cravings [42]. It also seems that tailoring nutritional interventions to hormonal fluctuations may improve PMS symptoms [148]. 

Individualized nutritional therapy requires ongoing monitoring and periodic adjustments to the changing needs of those suffering from PMS.

## 6. Potential Impact of PMS and PMDD on Quality of Life, Interpersonal Relationships, and Work Productivity

Numerous observational studies have shown the adverse impact of PMS on the physical and psychological domains of quality of life [149,150,151,152], its social domains [153,154,155], and interpersonal relationships [156,157]. PMS may also affect productivity and professional life during the luteal phase and the post-episode period [158]. A significant correlation between PMS severity and several factors, including reduced presenteeism, intention to reduce working hours, and increased absenteeism from work, was found. Importantly, due to feelings of embarrassment, on rare occasions, women may choose to request sick leave and disclose their PMS symptoms, primarily because of concerns about the appropriateness of taking time off work [159]. A large case-control study showed a higher prevalence of productivity-related impairments in the PMS group than in the group without PMS [160]. PMS also affects sleep quality. A systematic review and meta-analysis showed the adverse effects of PMS on various aspects of sleep, including satisfaction, alertness, efficiency, and duration [161]. In addition, some studies have found an increased risk of suicide in women with PMS and PMDD [162]. 

The selected studies that assessed the adverse outcomes of PMS are presented in Table 5.

## 7. Future Direction 

Diet appears to be an important factor modulating the risk of development and severity of PMS symptoms. However, studies assessing the effects of macro- and micronutrients on the development of PMS and the severity of its symptoms are limited and are of insufficient quality. Further high-quality studies are needed to confirm the impact of diet on PMS e, in particular, double-/triple-blind placebo-controlled RCTs with a follow-up of large cohorts. In addition, confounding factors including stress, interpersonal relationships, and meal supervision should be considered. This will allow for the formulation of more effective nutritional recommendations for women with PMS.

## 8. Conclusions

Diet seems to be an important factor in the development of PMS and the modulation of its symptoms. A diet consisting of unprocessed, fresh foods and limiting simple carbohydrates, fats, salt, and alcohol intake may prevent the development of PMS and reduce the severity of its symptoms. However, further studies are necessary to formulate clear dietary recommendations. In addition, the benefits of individualized micronutrients and vitamin supplementation in the treatment of PMS are suggested. However, it should be emphasized that large randomized placebo-controlled trials in racially diverse populations are needed to formulate definitive and personalized recommendations regarding the supplementation of micronutrients and vitamins in women with PMS.

## Figures and Tables

**Table 1:** The studies assessed the associations between macronutrient intake and premenstrual syndrome (PMS)/Premenstrual Dysphoric Disorder (PMDD).

Author	Methods of Diet Analysis	Characteristics of Population	Type of Study	Results
Hashim, Mona et al. [48], 2019	Self-administered, semi-quantitative food-frequency questionnaire (FFQ)	300 Arabian women (95% with at least one PMS symptom)	cross-sectional study	High fat, sugar, and salt intake was associated with an increased risk of physical symptoms of PMS (OR = 3.2, 95% CI 1.4–7.3; *p* < 0.05)
Farasati et al. [49] 2015	Self-administered, semi-quantitative food-frequency questionnaire (FFQ)	320 Iranian nurses (160 with and 160 without PMS)	case-control study	A significant association between the Western dietary pattern and PMS was shown. In participants in the second and third quartiles of the Western dietary pattern, PMS occurred more frequently than in women in the first quartile (OR = 2.53; 95% CI = 1.18, 5.43; OR = 4.39; 95% CI 1.97, 9.81, respectively). The relationship remained significant after adjustment for age, BMI, menstrual cycle status, physical activity and energy intake (OR = 2.53; 95% CI 1.18, 5.43 and OR = 4.39; 95% CI 1.97, 9.81, respectively)
Thakur et al. [51], 2022	3-day food diary including one weekend day and 70 food items FFQ	330 Indian women (46.9% with mild PMS, 31.5% with moderate PMS, 8.3% with severe PMS, and 13.3% without PMS symptoms)	observational study	Lactovegetarians and ovolactovegetarian women experienced milder PMS symptoms than women who consumed a non-vegetarian diet. The energy consumption and protein, dietary fiber, calcium, iron, vitamin C, and vitamin B12 levels were lower than the recommended dietary allowance (RDA). Carbohydrate intake was slightly higher, and fat was significantly higher than that of RDA. A significant correlation between PMS and the consumption of sweets, fried savory foods, and fast foods was found. An inverse association between oilseed consumption and PMS was shown.
Taheri, et al. [54], 2023	147 food items FFQ	223 Iranian women (25% with PMS)	cross-sectional	Total fat and sugar intake were associated with PMS.
MoradiFili, et al. [55], 2020	Self-administered, semi-quantitative food-frequency questionnaire (FFQ)	559 (225 Iranian women with PMS and 334 healthy controls aged 20–45 years	case-control study	A significant association was observed between the Western dietary pattern (high intake of fast foods, soft drinks, and processed meats) and PMS. PMS occurred frequently in participants in the highest tertile of the Western diet patterns (OR = 1.49; 95% CI 1.01, 3.52), *p* < 0.001). A negative correlation between healthy (rich in dried fruits, condiments, and nuts) and traditional (rich in eggs, tomato sauce, fruits, and red meat) dietary patterns and PMS was found (OR = 0.31; 95% CI: 0.17, 0.72, *p* = 0.02; OR = 0.33; 95% CI: 0.14, 0.77, *p* = 0.01, respectively).
Houghton et al. [47], 2018	131-item FFQ at baseline and every four years during follow-up	116,429 American nurses aged 25–42 years (during observation 4108 new diagnoses of PMS)	prospective cohort study with 14 years of follow-up	Maltose intake at 14 years of age was linearly associated with the risk of PMS (*p* for trend = 0.005). The highest intake (median = 3.0 g/day) was associated with a 45% higher risk of developing PMS than the lowest intake (median = 1.2 g/day) (95% CI = 1.11–1.88).
Houghton et al. [56], 2017	131-item semi-quantitative FFQ	3660 American women baselines without PMS aged 25–42 years (during observation 1234 with and 2426 without PMS)	prospective cohort study with 14 years of follow-up	High SFA intake, especially stearic acid, was associated with lower PMS risk (relative risk (RR) quintile 5 (median = 28 · 1 g/d) vs. quintile 1 (median = 15 · 1 g/d) = 0 · 75; 95% CI 0 · 58, 0 · 98; p for trend = 0 · 07).

**Table 2:** The studies assessed the associations between micronutrient intake and premenstrual syndrome (PMS)/Premenstrual Dysphoric Disorder (PMDD).

Author	Methods	Characteristics of Population	Type of Study	Results
Quaglia et al. [44], 2023	food diaries	30 Italian women aged 19–49 years (16 with PMS)	observational study	copper intake was significantly higher in women with PMS.
Taheri et al. [54], 2023	147-item FFQ	223 Iranian women (25% with PMS)	retrospective cross-sectional study	sodium intake was associated with an increased risk of the development of PMS
Thys-Jacobs et al. [60], 1998	Supplementation of 1200 mg calcium carbonate or placebo	466 premenopausal women (231 patients treated with calcium and 235 with the placebo)	A Multicenter clinical trial	no significant differences between groups in the mean screening symptom complex score of the luteal, menstrual, or intermenstrual phase of the menstrual cycle were found A significantly lower mean symptom complex score was observed in the group treated with calcium for both the second (*p* = 0.007) and third (*p* < 0.001) treatment cycles
Saeedian et al. [59], 2015	24-h food recall questionnaire	62 Iranian young women (31 with PMS)	case-control study	calcium, magnesium and potassium intake were lower in the PMS group
Chocano-Bedoya et al. [61], 2013	food frequency questionnaires completed in 1991, 1995, and 1999	1057 American women with PMS and 1968 without PMS at baseline	prospective case-control study with 10 years of follow-up	The highest quintile of nonheme iron intake was associated with a 64% RR of PMS (95% CI 0.44, 0.92; *p* for trend = 0.04). The highest quintile of potassium intake was associated with 146% RR of PMS (95% CI: 0.99, 2.15; *p* for trend = 0.04).
Ahmadi et al. [63], 2023	220 mg elemental zinc or placebo supplementation daily for 24 weeks	69 young Iranian women (35 with PMS)	randomized clinical trial	Zinc supplementation significantly decreases physical and psychological symptoms of PMS.
Jafari et al. [64], 2020	30 mg zinc gluconate or placebo supplementation daily for 12 weeks	60 Iranian women aged 18–30 years with PMS	randomized clinical trial	Zinc supplementation significantly decreases physical (*p* < 0.05) and psychological (*p* < 0.001) symptoms of PMS.

**Table 3:** The studies assessed the associations between vitamin intake and premenstrual syndrome (PMS)/Premenstrual Dysphoric Disorder (PMDD).

Author	Methods	Characteristics of Population	Type of Study	Results
Chocano-Bedoya et al. [65], 2011	FFQ at the baseline and twice after 4 and 8 years	3025 American women without PMS at the baseline (1968 controls and 1057 with PMS after 10 years)	case-control study	An inverse relationships between the occurrence of PMS and thiamine and riboflavin intake were found.
Retallick-Brown et al. [66], 2020	Supplementation with 80 mg daily vitamin B6 or micronutrients during three menstrual cycles	78 women with PMS	pilot randomized treatment-controlled trial	72% of the micronutrient and 60% of the vitamin B6 group achieved complete remission in PMS symptoms.
Abdollahifard et al. [68], 2014	Supplementation with 100 mg daily thiamine or placebo one pill in the morning and one pill at night or placebo during one week before menstruation for three consecutive menstrual cycles	80 Iranian women with PMS aged 18–30 years	double-blind placebo-controlled clinical trial randomization 1:1	Thiamine supplementation reduces by 35.08% mental symptoms and by 21.2% physical symptoms of PMS.
Samieipour et al. [69], 2016	Supplementation with 100 mg thiamine or 500 mg calcium carbonate or both or placebo once daily from one week before menstruation to 4 days after menstruation for two consecutive menstrual cycles	264 Iranian women with PMS aged 18–30 years	randomized controlled trial randomization 1:1:1:1	Supplementation with thiamine decreased PMS symptoms more than placebo. Supplementation with calcium decreased PMS symptoms more than thiamine and placebo. The reduction symptoms of PMS in the group supplemented with both thiamine and calcium were higher than in the other groups.
Bertone-Johnson et al. [70], 2005	assessment of vitamin D intake using FFQ at the baseline and during 4 and 8 years of the follow-up	3025 American women aged 27–44 years without PMS at baseline (1968 controls and 1057 with PMS)	prospective case-control study with 10 years of follow-up	High vitamin D intake was associated with a lower risk of the development of PMS [RR 0.59 (95% CI, 0.40–0.86).
Bertone-Johnson et al. [72], 2010	FFQ	44 American women aged 18–30 years meeting standard criteria for PMS and 46 women meeting control criteria	cross-sectional study	No significant inverse relationship between vitamin D intake from food sources and the severity of PMS symptoms was found.
Rajaei et al. [73], 2016	Levels of 25 hydroxy-vitamin D3 (25OHD) were determined by ELISA in the luteal phase	82 Iranian women aged 18–40 years	case-control study	No significant relationship between the severity of PMS symptoms and the vitamin D levels was shown.
Bahrami et al. [74], 2018	Supplementation with nine high-dose vitamin D (50,000 IU/week of cholecalciferol)	897 Iranian adolescent girls (14.9% with PMS)	clinical trial	The occurrence of PMS after the intervention decreased from 14.9% to 4.8% (*p* < 0.001).
Tartagni et al. [75], 2016	Treatment with 200,000 IU first, followed by 25,000 IU every 2 weeks, or placebo for 4 months.	158 Italian girls (78 placebo group, 80 vitamin-D group)	randomized controlled clinical trial	Treatment with vitamin D reduces significantly anxiety, irritability, ease of crying and sadness as well as significantly improves disturbed interpersonal relationships.
Karimi et al. [76], 2018	Four groups: cognitive–behavioral therapy; supplementation 500 mg calcium and 200 IU vitamin D; cognitive–behavioral therapy + supplementation 500 mg calcium and 200 IU vitamin D; the lack of intervention for 8 weeks	40 Iranian women aged 22–48 years	quasi-experimental randomization 1:1:1:1	Significant improvement in PMS symptoms in the group treated with calcium plus vitamin D together with CBT than in the other groups.
Khajehei et al. [77], 2009	Three groups: treated with 5 mg of dydrogesterone or supplemented with 500 mg of calcium plus 200 mg of vitamin D, or placebo twice daily from the 15th to the 24th day of the cycle for 2 menstrual cycles	180 Iranian young women with PMS	randomized, double-blind, placebo-controlled study	Treatment with dydrogesterone or calcium plus vitamin D similarly decreased severity of PMS symptoms (by 4.64% and 4.20%, respectively) and placebo by 3.42%. The greatest effects were observed in loss of concentration, disturbed interpersonal relationships, anxiety, and arthralgia.
Dadkhah et al. [78], 2016	Supplementation with 200 mg of vitamin D, or 100 mg of vitamin E, or a placebo each day during 2 consecutive menstrual cycles	86 Iranian women with PMS aged 15–45 years	randomized controlled trial	The mean score of the syndrome significantly decreased in all three groups. However, there were no differences between groups.

**Table 4:** The studies assessed the associations between other nutrient intake and premenstrual syndrome (PMS)/Premenstrual Dysphoric Disorder (PMDD).

Author	Methods	Characteristics of Population	Type of Study	Results
Schmidt et al. [80], 2018	Supplementation with 400 mg lecithin- phosphatidylserine and 400 mg phosphatidic acid or placebo for 3 menstrual cycles	40 women with PMS aged 18–45 years	randomized controlled trial	The study supplements significantly more than placebo reduce PMS physical symptoms severity and depression.
Rossignol et al. [82], 1985	caffeine-containing beverages including coffee, tea and cola intake	295 American college sophomores	questionnaire-based study	A relationship between the consumption of caffeine-containing beverages and the occurrence and severity of PMS was found.
Rossignol et al. [87], 1990	caffeine-containing beverages including coffee, tea and cola intake	841 American students	questionnaire-based study	dose-dependent relationship between the consumption of caffeine-containing beverages and the occurrence of PMS (OR = 1.3 for consumers of one cup of caffeine-containing beverages per day, OR = 7.0 for consumers of 8 to 10 cups per day)
Rasheed et al. [89], 2003	Participants indicated the type of caffeinated beverage consumed from a list including cola drinks, tea, cocoa chocolates and coffee, including the Arab coffee “Gahwa”.	464 Saudi Arabian women aged 17–27 years	questionnaire-based study	A relationship between the consumption of caffeinated, coffee (especially over 7 cups per week) and the severity of PMS symptoms was found.
Purdue-Smithe et al. [93], 2016	Caffeine, coffee, and tea intake was assessed using food-frequency questionnaires every 4 years	1234 women with PMS, 2426 controls	case-control study nested within the prospective Nurses’ Health Study II	There was no association between the risk of the development of PMS or its specific symptoms and total caffeine intake.
Sayegh et al. [94], 1995	Supplementation with the specially formulated carbohydrate-rich beverage compared with two other isocaloric	24 women with PMS	double-blind, crossover study	The reduction of psychological symptoms of PMS and appetite can be obtained by specially formulated carbohydrate-rich beverage intake

**Table 5:** These studies assessed the impact of PMS on quality of life, interpersonal relationships, and work productivity.

Author	Cohort	Prevalence of PMS (%)	Adverse Outcomes
Al-Shahrani et al. [149], 2021	338 female Saudi Arabian medical students	64.9%	PMS significantly influenced daily activities related to quality of life and homework as well as their learning environment.
Kahyaoglu et al. [150], 2016	134 Turkish nurses	38.1%	All of the WRQoL subscale scores except stress at work were significantly lower in the group with than without PMS.
İşik et al. [151], 2016	608 Turkish health sciences students	84.5%	The quality of life decreases with the severity of PMS.
Farrokh-Eslamlou et al. [152], 2015	142 Iranian female medical students	39.4% including 60.6% with mild, 25.1% with moderate and 14.2% with severe PMS	The quality of life score means in mental health (*p* = 0.02) and environmental health decreases as the PMS score average increases. PMS has adverse effects on academic performance and related quality of life.
Câmara et al. [153], 2017	801 Brasilian women aged 18 years and over	39.7% with moderate to severe PMS 16.5% with PMDD	Physical, psychological and social domain quality of life decreased with the severity of PMS/PMDD.
Victor et al. [154], 2019	642 Brasilian female students aged 18–24 years	49.9% including 23.3% with mild PMS and 26.6% with PMDD	Both physical and mental domains of WHOQL-Bref were significantly lower in women with mild PMS and PMDD than without PMS. Social relationships and environmental domains were significantly lower in women with mild PMS than in those without PMS.
Jaber et al. [155], 2022	179 Jordanian women aged 20–30 years	88%	PMS affects daily activities, satisfaction with general appearance and weight and relationships with family members and other people.
Karimiankakolaki et al. [156], 2019	246 Iranian women aged 15–49 years	87.4%	The effect of PMS on daily life was stronger in terms of relationships with family. In addition, marital dissatisfaction was higher among women with PMS than without PMS.
Hardy et al. [158], 2021	125 working women from UK	40% with moderate to severe symptoms of PMS	Severe PMS symptoms were significantly associated with poor presenteeism, intention to reduce working hours, and higher work absence (time off work, being late, leaving early). Moderate/severe symptoms were significantly associated with poorer work-life balance, lower levels of psychological resilience, higher perceived work demands, and less control over work.
Heinemann et al. [159], 2010	822 German women aged 15–45 years	56.7% with mild PMS 30.25% with moderate to severe PMS 4.9% with PMDD	Employed women with moderate to severe PMS/PMDD had a higher rate of productivity impairment than those with no perceived symptoms/mild PMS (adjusted OR, 3.12; 95% CI: 1.75–5.57). Women with moderate to severe PMS/PMDD had a higher rate of absenteeism (>8 h per cycle; 14.2% vs. 6.0%).
Jeon et al. [160], 2023	a systematic review of 35 studies including 26,867 women		PMS was associated with sleep disturbances including satisfaction, alertness during waking hours, efficiency and duration.
Prasad et al. [161], 2021	systematic review and meta-analysis of 13 studies including 12,929 women		Women with PMDD are almost seven times at higher risk of suicide attempt (OR: 6.97; 95% CI: 2.98–16.29, *p* < 0.001) and almost four times as likely to exhibit suicidal ideation (OR: 3.95; 95% CI: 2.97–5.24, *p* < 0.001). Women with PMS have also an increased risk of suicidal ideation (OR: 10.06; 95% CI: 1.32–76.67, *p* = 0.03), but not suicide attempts (OR: 1.85; 95% CI: 0.77–4.46, *p* = 0.17)
Yan et al. [162], 2021	meta-analysis of 6 studies including 8532 women		PMDD was associated with an increased risk of: - suicidal ideation (OR = 2.34, 95% CI: 1.50–3.18, *p* = 0.99), - experiencing suicide attempt (OR = 2.13, 95% CI: 1.05–3.21, *p* = 0.81), - suicidal plan (OR = 2.24, 95% CI: 1.03–3.45, *p* = 0.96).

## Data Availability

Not applicable.
